# Crystal structure of [1,3-bis­(2,4,6-tri­methyl­phen­yl)imidazolidin-2-yl­idene]di­chlorido­(2-{[(2-methoxyeth­yl)(meth­yl)amino]­meth­yl}benzyl­idene)ruth­en­ium

**DOI:** 10.1107/S2056989023010381

**Published:** 2024-01-01

**Authors:** Gunay Z. Mammadova, Zeliha Atioğlu, Mehmet Akkurt, Mikhail S. Grigoriev, Nikita S. Volchkov, Asmet N. Azizova, Ajaya Bhattarai, Alexandra S. Antonova

**Affiliations:** a Organic Chemistry Department, Baku State University, Z. Xalilov Str. 23, Az 1148 Baku, Azerbaijan; b Department of Aircraft Electrics and Electronics, School of Applied Sciences, Cappadocia University, Mustafapaşa, 50420 Ürgüp, Nevşehir, Türkiye; c Department of Physics, Faculty of Sciences, Erciyes University, 38039 Kayseri, Türkiye; d Frumkin Institute of Physical Chemistry and Electrochemistry, Russian Academy of Sciences, Leninsky pr. 31, bld. 4, Moscow 119071, Russian Federation; e Organic Chemistry Department, Faculty of Science, RUDN University, Miklukho-Maklaya St., 6, Moscow 117198, Russian Federation; f Department of Synthesis of Biologically Active Compounds, Scientific Research Center, Azerbaijan Medical University, Samed Vurgun St. 167, Az 1022 Baku, Azerbaijan; g Department of Chemistry, M.M.A.M.C (Tribhuvan University) Biratnagar, Nepal; University of Neuchâtel, Switzerland

**Keywords:** crystal structure, ruthenium catalyst, Hoveyda–Grubbs catalyst, olefin metathesis

## Abstract

The title compound, [RuCl_2_(C_33_H_43_N_3_O)], is an example of a new generation of *N*,*N*-dialkyl ruthenium catalysts with an N—Ru coordination bond as part of a six-membered chelate ring. The Ru atom has an Addison τ parameter of 0.244, which indicates a geometry inter­mediate between square-based pyramidal and trigonal–bipyramidal. The complex shows the usual *trans* arrangement of the two chlorides, with Ru—Cl bond lengths of 2.3515 (8) and 2.379 (7) Å, and a Cl—Ru—Cl angle of 158.02 (3)°. One of the chlorine atoms and the atoms of the 2-meth­oxy-*N*-methyl-*N*-[(2-methyl­phen­yl)meth­yl]ethane-1-amine group of the title complex display disorder over two positions in a 0.889 (2): 0.111 (2) ratio.

## Abstract 2

The title compound [RuCl_2_(C_33_H_44_N_3_O)] is an example of a new generation of *N*,*N*-dialkyl metallocomplex ruthenium catalysts with an N→Ru coordination bond in a six-membered chelate ring.

## 1. Chemical context

Over the past decades, significant progress has been made in the conceptualization of methodology of organometallic catalytic systems for olefin metathesis (for selected reviews and books on the topic, see: Grela, 2014 ▸; Ogba *et al.*, 2018 ▸; Mukherjee *et al.* 2018 ▸; Tsedalu, 2021 ▸; Copéret *et al.*, 2021 ▸). This has made it possible to successfully overcome some of the limitations that initially prevented the integration of ruth­enium catalysts in both laboratory practice and industry. The list of such areas includes production of bioactive substances with a desired selectivity of the resulting double bond, obtaining highly functionalized organic compounds, and the synthesis of new materials including polymers (Pederson *et al.*, 2002 ▸; Kozłowska *et al.*, 2014 ▸; Eivgi *et al.*, 2020 ▸). Complexes including a six-membered chelate ruthenium ring are effective catalysts for various types of olefin metathesis reactions (Polyanskii *et al.*, 2019*a* ▸,*b* ▸; Kumandin *et al.*, 2020 ▸, 2023 ▸; Antonova *et al.*, 2020 ▸; Vasilyev *et al.*, 2023 ▸). On the other hand, the catalytic activity of metal complexes is dictated by the ligands, while the coordination environment of the metal center and ligands can be decorated by attaching different non-covalent bond donor or acceptor substituents for the regulation of the structure and the reactivity of the catalysts (Gurbanov *et al.*, 2022*a* ▸,*b* ▸; Mahmoudi *et al.*, 2017*a* ▸,*b* ▸; Mahmudov *et al.*, 2013 ▸, 2023 ▸). This work proposes a method for obtaining a new chelate complex from the commercially available precursors [RuCl_2_(1,3-bis­(2,4,6-tri­methyl­phen­yl)imidazoline-2-yl­idene)(3-phenyl­indenylide-1-ene)(pyridine)] and styrene 2-meth­oxy-*N*-methyl-*N*-(2-vinyl­benz­yl)ethan-1-amine. By X-ray analysis, it was proved that this complex is a *trans*-isomer, relative to the arrangement of the two chlorides. 
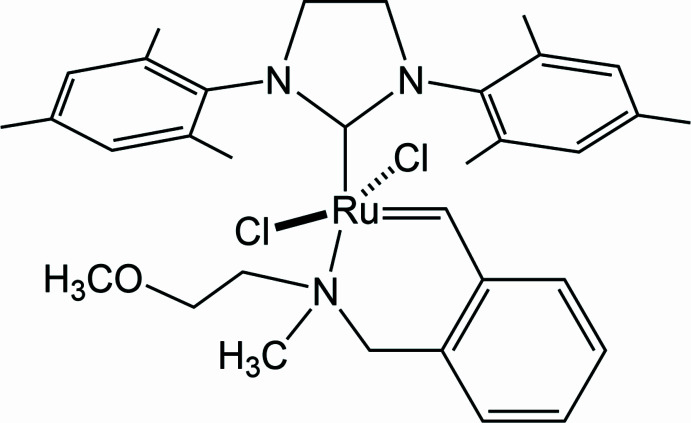


## 2. Structural commentary

**Figure 1:**
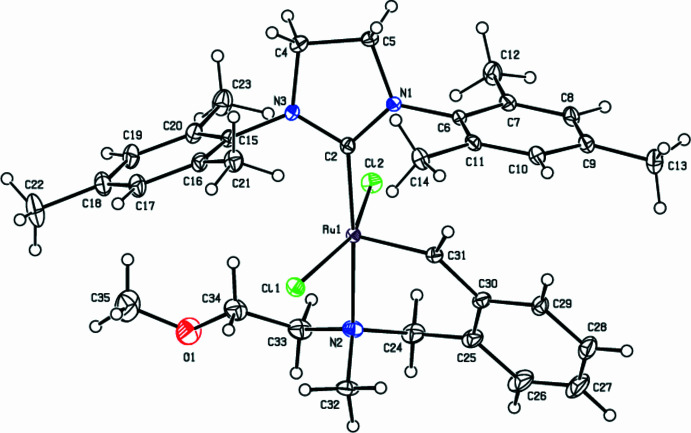
The mol­ecular structure of the title complex with displacement ellipsoids for the non-hydrogen atoms drawn at the 30% probability level. Only the major component of the disorder is shown for clarity.

**Figure 2:**
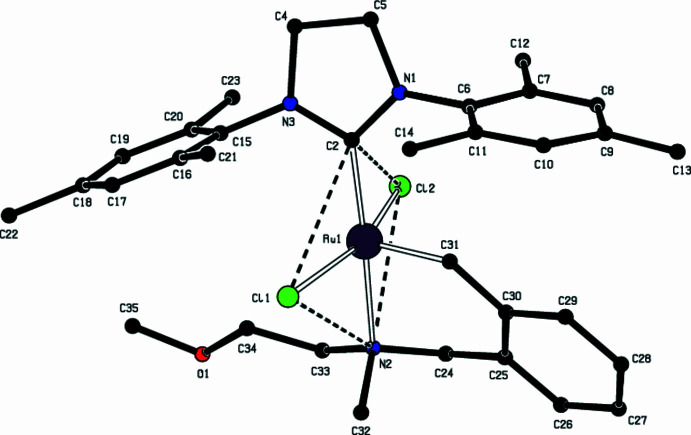
A view of the coordination geometry about the Ru atom, which lies between square-based pyramidal and trigonal–bipyramidal for major disorder component Cl2.

The Ru atom in the title compound is penta­coordinated to two C, one N and two Cl atoms (Fig. 1 ▸, Table 1 ▸). The Addison parameter is used to describe the distortion of the coordination geometry and is defined as τ (difference between two largest angles / 60 for five-coordinated metal centers), allowing the distinction between trigonal–bipyramidal (ideally τ = 1) and square-pyramidal (ideally τ = 0) geometries (Addison *et al.*, 1984 ▸). For the title complex, τ = 0.244, which is between these two geometries (Fig. 2 ▸), [τ = 0.09 for minor disorder Cl2′ and the coordination geometry is closer to square pyramidal]. The dihedral angle between the planes of the tri­methyl­phenyl rings is 26.34 (10)°. The complex shows the usual *trans* arrangement of the two chlorides, with Ru—Cl bond lengths of 2.3515 (8) and 2.379 (7) Å, and a Cl—Ru—Cl angle of 158.02 (3)°. The bond lengths and angles about the Ru atom are in good agreement with those reported for similar compounds (see *Database survey* section).

## 3. Supra­molecular features

**Figure 3:**
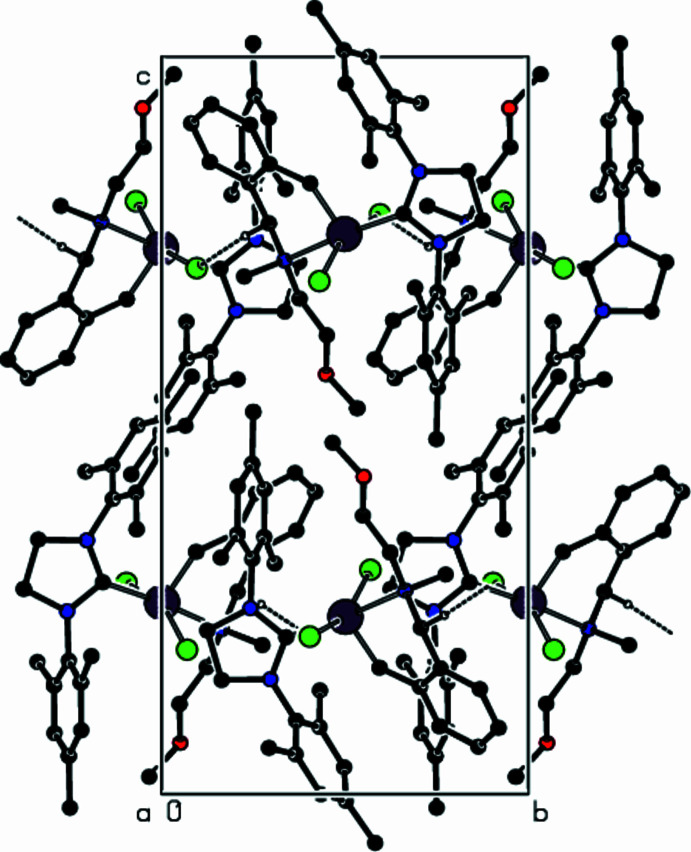
The packing of the title complex, showing the C—H⋯Cl inter­actions along the *a*-axis direction as dashed lines. For clarity, only the major component of the disorder and the hydrogen atoms involved in the bonding are shown.

The crystal structure of the title complex includes intra- and inter­molecular C—H⋯Cl inter­actions (Tables 2 ▸ and 3 ▸). In the ten intra­molecular C—H⋯Cl inter­actions, the H⋯Cl distances vary from 2.56 to 2.94 Å, while the C—H⋯Cl angles vary from 110 to 129°. The inter­molecular C—H⋯Cl inter­actions in the title complex are shown in Fig. 3 ▸. A weak intra­molecular C—H⋯π inter­action is also observed.

## 4. Database survey

The compounds [1,3-bis(2,4,6-tri­methyl­phen­yl)imidazolidin-2-yl­idene]-di­chloro-{2-[1-(di­methyl­amino)­eth­yl]benzyl­idene}ruthenium (CSD refcode TITTUO; Polyanskii *et al.*, 2019*a* ▸), *cis*-di­chlorido-(1,3-dimesitylimidazolidin-2-yl­idene)(2-formyl­benzyl­idene-*C*,*O*)ruthenium diethyl ether solvate (DULVOW; Slugovc *et al.*, 2010 ▸) and *cis*-(SPY-5-34)-di­chloro­(4,5-di­hydro-1,3-dimesitylimidazol-2-yl­idene)(2-formyl­benzyl­idene-*C*,*O*)ruthenium (XACYOQ; Slugovc *et al.*, 2004 ▸) show similar metal-atom geometries to the title compound.

In XACYOQ, mol­ecules are linked by C—H⋯Cl, C—H⋯π and π–π-stacking inter­actions. In the crystal structures of TITTUO and DULVOW, inter­molecular π–π stacking is an important factor and these inter­actions form a framework-like structure containing channels that extend along the *b* and *c* axes, respectively (Samojłowicz *et al.*, 2009 ▸).

## 5. Synthesis and crystallization

In a Schlenk flask, ruthenium precursor complex [RuCl_2_(1,3-bis­(2,4,6-tri­methyl­phen­yl)imidazoline-2-yl­idene)(3-phenyl­indenylide-1-ene)(pyridine)] (200 mg, 0.26 mmol, 1.0 equiv.) was dissolved in dry toluene (4 mL) under an argon atmosphere. Then the styrene (0.31 mmol, 1.2 equiv.) was added in an argon stream, after that the flask was sealed with a screw cap and heated at 353 K for 1 h. The reaction mixture was placed in a freezer (253 K) for 30 min. The precipitate was filtered off and washed sequentially with hexane (3 × 5 mL) and methanol (3 × 3 mL), both cooled to 253 K, to give the title complex as a green powder after drying under vacuum for 2 h. A single crystal was obtained by slow crystallization from a hexa­ne/chloro­form mixture at 298 K.

Green powder, 104 mg, 0.41 mmol, 60%, *R*_*f*_ = 0.85 (Sorbfil plates for thin-layer chromatography, EtOAc: hexane, 1:2); mp: 481.1–483.5 K (dec.).

^1^H NMR (700.2 MHz, CDCl_3_, 298 K) δ 18.73 (*s*, 1H, CH=Ru), 7.46 (*dd*, *J* = 1.2, 7.5 Hz, 1H, H-4-C_6_H_4_), 7.10–6.96 (*m*, 6H, H-3-C_6_H_4_, H-5-C_6_H_4_, H-Mes), 6.57 (*d*, *J* = 6.7 Hz, 1H, H-6-C_6_H_4_), 5.36 (*d*, *J* = 8.1 Hz, 1H, CH_2_N-*A*), 4.04 (*br.s*, 4H, NCH_2_CH_2_N), 5.36 (*br.s*, 1H, CH_2_N-*B*), 3.15 (*s*, 1H, NC**H**_2_CH_2_OMe-*A*), 3.09 (*s*, 3H, NCH_3_), 3.01 (*m*, 2H, NCH_2_C**H**_2_O), 2.58 (*s*, 6H, Mes-Me), 2.41 (*s*, 12H, Mes-Me), 2.17 (*s*, 1H, NC**H**_2_CH_2_OMe-*B*), 1.80 (*s*, 3H, OCH_3_).

^13^C NMR (176.1 MHz, CD_2_Cl_2_, 298 K) δ 314.0, 212.6, 148.1, 139.0 (4C), 138.5, 136.7 (2C), 133.3, 130.9 (2C), 129.8 (2C), 129.6 (2C), 128.7, 128.4, 126.8, 69.5 (2C), 66.0, 60.9, 58.3, 51.7, 46.2, 21.2 (3C), 19.6 (3C).

IR ν_max_/cm^−1^ (KBr pellets): 3438, 3258, 2910, 1953, 1632, 1610, 1485, 1440, 1410, 1280, 1264, 1073, 1011, 953, 940, 850, 804, 745. HRMS (ESI-TOF): calculated for C_33_H_43_ClNORu [*M* - Cl]^+^ 634.2133; found 634.2138.

## 6. Refinement

Crystal data, data collection and structure refinement details are summarized in Table 4 ▸. All C-bound H atoms were included in the refinement using the riding-model approximation with C—H distances of 0.95–0.99 Å, and with *U*_iso_(H) = 1.2 or 1.5*U*_eq_(C). The measurements of the 0 0 2, 1 0 1, 0 1 1, 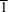 0 1 and 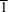 1 2 reflections were affected by the beam stop and they were therefore excluded from the refinement. The Cl2 chlorine atom and the atoms of the 2-meth­oxy-*N*-methyl-*N*-[(2-methyl­phen­yl)meth­yl]ethane-1-amine group of the title complex are disordered over two sites with refined occupancy factors of 0.889 (2) and 0.111 (2). SADI, SIMU, SAME and EADP instructions were used to refine disordered atoms.

## Supplementary Material

Crystal structure: contains datablock(s) I, shelx. DOI: 10.1107/S2056989023010381/tx2079sup1.cif

Structure factors: contains datablock(s) I. DOI: 10.1107/S2056989023010381/tx2079Isup2.hkl

CCDC reference: 2311600

Additional supporting information:  crystallographic information; 3D view; checkCIF report

## Figures and Tables

**Table 1:** Selected geometric parameters (Å, °)

Ru1—C31′	1.81 (3)	Ru1—N2	2.271 (2)
Ru1—C31	1.833 (4)	Ru1—Cl2	2.3515 (8)
Ru1—C2	2.0474 (18)	Ru1—Cl1	2.3519 (5)
Ru1—N2′	2.251 (15)	Ru1—Cl2′	2.379 (7)
			
C31′—Ru1—C2	100.5 (8)	C31′—Ru1—Cl2′	96.9 (15)
C31—Ru1—C2	97.33 (11)	C2—Ru1—Cl2′	78.3 (2)
C31′—Ru1—N2′	92.1 (8)	N2′—Ru1—Cl2′	90.2 (6)
C2—Ru1—N2′	163.8 (5)	Cl1—Ru1—Cl2′	158.4 (3)
C31—Ru1—N2	88.48 (11)	N3—C2—Ru1	121.14 (13)
C2—Ru1—N2	172.68 (8)	N1—C2—Ru1	131.33 (13)
C31—Ru1—Cl2	103.42 (13)	C32—N2—Ru1	115.62 (17)
C2—Ru1—Cl2	86.77 (5)	C24—N2—Ru1	108.54 (16)
N2—Ru1—Cl2	87.55 (7)	C33—N2—Ru1	110.59 (15)
C31′—Ru1—Cl1	104.7 (14)	C30—C31—Ru1	130.7 (2)
C31—Ru1—Cl1	98.02 (13)	C24′—N2′—Ru1	105.1 (11)
C2—Ru1—Cl1	95.36 (5)	C32′—N2′—Ru1	112.5 (13)
N2′—Ru1—Cl1	91.3 (6)	C33′—N2′—Ru1	111.4 (12)
N2—Ru1—Cl1	88.21 (7)	C30′—C31′—Ru1	130.8 (19)
Cl2—Ru1—Cl1	158.02 (3)		

**Table 2:** Hydrogen-bond geometry (Å, °)

*D*—H⋯*A*	*D*—H	H⋯*A*	*D*⋯*A*	*D*—H⋯*A*
C4—H4*A*⋯Cl1^i^	0.99	2.89	3.651 (2)	134
C5—H5*B*⋯Cl1^i^	0.99	2.83	3.622 (2)	137
C24—H24*A*⋯Cl2	0.99	2.56	3.257 (3)	127
C24—H24*B*⋯Cl2^ii^	0.99	2.72	3.693 (3)	167
C33—H33*B*⋯Cl2	0.99	2.87	3.399 (3)	114
C34—H34*A*⋯Cl1	0.99	2.94	3.570 (3)	123
C24′—H24*D*⋯Cl1	0.99	2.68	3.391 (19)	129
C32′—H32*D*⋯Cl2′	0.98	2.59	3.07 (2)	110
C32′—H32*F*⋯Cl2′^ii^	0.98	2.62	3.57 (2)	164
C4—H4*A*⋯Cl1^i^	0.99	2.89	3.651 (2)	134
C5—H5*B*⋯Cl1^i^	0.99	2.83	3.622 (2)	137
C24—H24*A*⋯Cl2	0.99	2.56	3.257 (3)	127
C24—H24*B*⋯Cl2^ii^	0.99	2.72	3.693 (3)	167
C33—H33*B*⋯Cl2	0.99	2.87	3.399 (3)	114
C34—H34*A*⋯Cl1	0.99	2.94	3.570 (3)	123
C24′—H24*D*⋯Cl1	0.99	2.68	3.391 (19)	129
C32′—H32*D*⋯Cl2′	0.98	2.59	3.07 (2)	110
C32′—H32*F*⋯Cl2′^ii^	0.98	2.62	3.57 (2)	164
C34′—H34*C*⋯*Cg*3	0.99	2.96	3.83 (2)	149

Symmetry codes: (i) 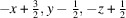 ; (ii) 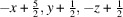 .

**Table 3:** Summary of short inter­atomic contacts (Å) in the title compound

Contact	Distance	Symmetry operation
H14*B*⋯H4*A*	2.46	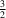 − *x*, 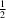 + *y*, 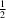 − *z*
*H34*D*⋯*H26	2.17	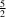 − *x*, − 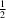 + *y*, 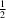 − *z*
*H29′⋯H12*C*	2.41	2 − *x*, 1 − *y*, −*z*
C9⋯H19	2.94	− 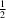 + *x*, 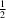 − *y*, − 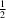 + *z*
*H28⋯*H35*B*	2.32	− 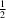 + *x*, 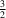 − *y*, − 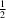 + *z*
*H26′⋯H23*A*	2.42	*x*, 1 + *y*, *z*
*H33⋯H22*B*	2.40	2 − *x*, 1 − *y*, 1 − *z*

**Table 4:** Experimental details

Crystal data	
Chemical formula	[RuCl_2_(C_21_H_26_N_2_)(C_12_H_17_NO)]
*M*_r_	669.67
Crystal system, space group	Monoclinic, *P*2_1_/*n*
Temperature (K)	100
*a*, *b*, *c* (Å)	13.6801 (13), 10.7078 (11), 22.035 (2)
β (°)	102.553 (4)
*V* (Å^3^)	3150.6 (5)
*Z*	4
Radiation type	Mo *K*α
μ (mm^−1^)	0.70
Crystal size (mm)	0.40 × 0.36 × 0.24

Data collection	
Diffractometer	Bruker Kappa APEXII area-detector diffractometer
Absorption correction	Multi-scan (*SADABS*; Krause *et al.*, 2015 ▸)
*T*_min_, *T*_max_	0.679, 0.746
No. of measured, independent and observed [*I* > 2σ(*I*)] reflections	54060, 9279, 7087
*R*_int_	0.049
(sin θ/λ)_max_ (Å^−1^)	0.707

Refinement	
*R*[*F* ^2^ > 2σ(*F* ^2^)], *wR*(*F* ^2^), *S*	0.032, 0.070, 1.02
No. of reflections	9279
No. of parameters	493
No. of restraints	460
H-atom treatment	H-atom parameters constrained
Δρ_max_, Δρ_min_ (e Å^−3^)	0.43, −0.58

Computer programs: *APEX3* and *SAINT* (Bruker, 2018 ▸), *SHELXT2016/6* (Sheldrick, 2015*a* ▸), *SHELXL2016/6* (Sheldrick, 2015*b* ▸), *ORTEP-3 for Windows* (Farrugia, 2012 ▸) and *PLATON* (Spek, 2020 ▸).

## supplementary crystallographic information

### Crystal data

**Table:**

[RuCl_2_(C_21_H_26_N_2_)(C_12_H_17_NO)]	*F*(000) = 1392
*M**_r_* = 669.67	*D*_x_ = 1.412 Mg m^−^^3^
Monoclinic, *P*2_1_/*n*	Mo *K*α radiation, λ = 0.71073 Å
*a* = 13.6801 (13) Å	Cell parameters from 9880 reflections
*b* = 10.7078 (11) Å	θ = 2.4–29.8°
*c* = 22.035 (2) Å	µ = 0.70 mm^−^^1^
β = 102.553 (4)°	*T* = 100 K
*V* = 3150.6 (5) Å^3^	Bulk, dark green
*Z* = 4	0.40 × 0.36 × 0.24 mm

### Data collection

**Table:**

Bruker Kappa APEXII area-detector diffractometer	7087 reflections with *I* > 2σ(*I*)
φ and ω scans	*R*_int_ = 0.049
Absorption correction: multi-scan (SADABS; Krause *et al.*, 2015)	θ_max_ = 30.2°, θ_min_ = 2.7°
*T*_min_ = 0.679, *T*_max_ = 0.746	*h* = −19→18
54060 measured reflections	*k* = −15→15
9279 independent reflections	*l* = −31→31

### Refinement

**Table:**

Refinement on *F*^2^	460 restraints
Least-squares matrix: full	Hydrogen site location: inferred from neighbouring sites
*R*[*F*^2^ > 2σ(*F*^2^)] = 0.032	H-atom parameters constrained
*wR*(*F*^2^) = 0.070	*w* = 1/[σ^2^(*F*_o_^2^) + (0.023*P*)^2^ + 2.0327*P*] where *P* = (*F*_o_^2^ + 2*F*_c_^2^)/3
*S* = 1.02	(Δ/σ)_max_ = 0.001
9279 reflections	Δρ_max_ = 0.43 e Å^−^^3^
493 parameters	Δρ_min_ = −0.58 e Å^−^^3^

### Special details

**Table:**

Geometry. All esds (except the esd in the dihedral angle between two l.s. planes) are estimated using the full covariance matrix. The cell esds are taken into account individually in the estimation of esds in distances, angles and torsion angles; correlations between esds in cell parameters are only used when they are defined by crystal symmetry. An approximate (isotropic) treatment of cell esds is used for estimating esds involving l.s. planes.

### Fractional atomic coordinates and isotropic or equivalent isotropic displacement parameters (Å^2^)

**Table:**

	*x*	*y*	*z*	*U*_iso_*/*U*_eq_	Occ. (<1)
Ru1	0.99437 (2)	0.50148 (2)	0.24032 (2)	0.01454 (4)	
Cl1	0.88778 (4)	0.56961 (5)	0.30419 (2)	0.02505 (10)	
Cl2	1.13328 (6)	0.40424 (9)	0.21357 (6)	0.02561 (19)	0.889 (2)
Cl2'	1.1181 (6)	0.3787 (9)	0.2073 (5)	0.02561 (19)	0.111 (2)
N1	0.87352 (12)	0.29680 (14)	0.15568 (7)	0.0189 (3)	
N3	0.92109 (12)	0.24251 (14)	0.25209 (7)	0.0195 (3)	
C2	0.91734 (13)	0.33997 (17)	0.21276 (8)	0.0166 (3)	
C4	0.88154 (17)	0.12448 (18)	0.22184 (9)	0.0287 (5)	
H4A	0.826509	0.091262	0.239829	0.034*	
H4B	0.934834	0.060604	0.225599	0.034*	
C5	0.84371 (16)	0.16398 (18)	0.15466 (9)	0.0252 (4)	
H5A	0.875913	0.114799	0.126348	0.030*	
H5B	0.770126	0.154553	0.141719	0.030*	
C6	0.83979 (14)	0.36710 (17)	0.09962 (8)	0.0194 (4)	
C7	0.89679 (16)	0.36545 (18)	0.05392 (9)	0.0240 (4)	
C8	0.86167 (16)	0.4348 (2)	−0.00026 (9)	0.0279 (4)	
H8	0.899326	0.435288	−0.031761	0.034*	
C9	0.77363 (16)	0.5029 (2)	−0.00927 (9)	0.0273 (4)	
C10	0.71724 (15)	0.49803 (19)	0.03628 (9)	0.0252 (4)	
H10	0.655536	0.542040	0.029767	0.030*	
C11	0.74867 (14)	0.43064 (18)	0.09096 (8)	0.0212 (4)	
C12	0.99271 (17)	0.2928 (2)	0.06261 (10)	0.0341 (5)	
H12A	0.977475	0.203641	0.056460	0.051*	
H12B	1.032465	0.306377	0.104767	0.051*	
H12C	1.030801	0.320867	0.032265	0.051*	
C13	0.73922 (19)	0.5826 (2)	−0.06635 (10)	0.0393 (6)	
H13A	0.752868	0.670631	−0.055536	0.059*	
H13B	0.667152	0.570936	−0.082252	0.059*	
H13C	0.775299	0.558099	−0.098330	0.059*	
C14	0.68862 (15)	0.4321 (2)	0.14068 (9)	0.0269 (4)	
H14A	0.627324	0.480823	0.126258	0.040*	
H14B	0.728444	0.470074	0.178534	0.040*	
H14C	0.670993	0.346359	0.149608	0.040*	
C15	0.94359 (15)	0.24718 (17)	0.31907 (8)	0.0207 (4)	
C16	0.86644 (15)	0.27982 (18)	0.34872 (9)	0.0236 (4)	
C17	0.88825 (17)	0.2812 (2)	0.41338 (9)	0.0308 (5)	
H17	0.837725	0.306289	0.434364	0.037*	
C18	0.98179 (18)	0.2470 (2)	0.44827 (10)	0.0347 (5)	
C19	1.05376 (17)	0.2080 (2)	0.41729 (10)	0.0332 (5)	
H19	1.116853	0.180996	0.440809	0.040*	
C20	1.03692 (16)	0.20690 (19)	0.35252 (9)	0.0265 (4)	
C21	0.76253 (15)	0.3087 (2)	0.31286 (10)	0.0292 (5)	
H21A	0.728626	0.230919	0.296992	0.044*	
H21B	0.766193	0.363879	0.277941	0.044*	
H21C	0.724905	0.350150	0.340223	0.044*	
C22	1.0032 (2)	0.2503 (3)	0.51858 (11)	0.0548 (8)	
H22A	1.033192	0.170881	0.535167	0.082*	
H22B	0.940564	0.263588	0.532379	0.082*	
H22C	1.049749	0.318708	0.533720	0.082*	
C23	1.11778 (17)	0.1615 (2)	0.32081 (11)	0.0373 (5)	
H23A	1.106250	0.073384	0.309524	0.056*	
H23B	1.183329	0.170749	0.349152	0.056*	
H23C	1.116202	0.211009	0.283198	0.056*	
N2	1.09671 (18)	0.6647 (2)	0.27615 (12)	0.0244 (5)	0.889 (2)
C24	1.14556 (18)	0.7073 (2)	0.22554 (12)	0.0311 (6)	0.889 (2)
H24A	1.180705	0.635577	0.211559	0.037*	0.889 (2)
H24B	1.196507	0.771195	0.242478	0.037*	0.889 (2)
C25	1.07400 (18)	0.7610 (2)	0.17056 (12)	0.0290 (5)	0.889 (2)
C26	1.1015 (2)	0.8680 (2)	0.14238 (14)	0.0432 (7)	0.889 (2)
H26	1.163691	0.906832	0.159698	0.052*	0.889 (2)
C27	1.0406 (3)	0.9191 (3)	0.08989 (17)	0.0488 (8)	0.889 (2)
H27	1.061284	0.991924	0.071474	0.059*	0.889 (2)
C28	0.9496 (3)	0.8640 (3)	0.06428 (15)	0.0403 (8)	0.889 (2)
H28	0.907683	0.898325	0.028052	0.048*	0.889 (2)
C29	0.9199 (2)	0.7582 (3)	0.09186 (13)	0.0276 (6)	0.889 (2)
H29	0.857368	0.720626	0.073949	0.033*	0.889 (2)
C30	0.9797 (4)	0.7052 (3)	0.14538 (14)	0.0226 (6)	0.889 (2)
C31	0.9409 (2)	0.5948 (3)	0.17136 (18)	0.0178 (7)	0.889 (2)
H31	0.877511	0.566781	0.148749	0.021*	0.889 (2)
C32	1.04729 (19)	0.7748 (2)	0.29788 (13)	0.0299 (6)	0.889 (2)
H32A	1.095373	0.843683	0.307309	0.045*	0.889 (2)
H32B	1.023770	0.752437	0.335407	0.045*	0.889 (2)
H32C	0.990211	0.800737	0.265227	0.045*	0.889 (2)
C33	1.18122 (18)	0.6234 (2)	0.32777 (13)	0.0321 (6)	0.889 (2)
H33A	1.218149	0.698051	0.346998	0.039*	0.889 (2)
H33B	1.228189	0.571875	0.310011	0.039*	0.889 (2)
C34	1.1470 (2)	0.5491 (3)	0.37763 (13)	0.0355 (6)	0.889 (2)
H34A	1.086385	0.587242	0.387542	0.043*	0.889 (2)
H34B	1.131107	0.462151	0.363592	0.043*	0.889 (2)
O1	1.2275 (2)	0.5512 (2)	0.43055 (14)	0.0521 (7)	0.889 (2)
C35	1.2131 (3)	0.4664 (4)	0.47665 (16)	0.0668 (11)	0.889 (2)
H35A	1.147148	0.480349	0.486017	0.100*	0.889 (2)
H35B	1.265216	0.478992	0.514398	0.100*	0.889 (2)
H35C	1.216949	0.380769	0.461615	0.100*	0.889 (2)
N2'	1.1124 (14)	0.6405 (16)	0.2836 (8)	0.027 (3)	0.111 (2)
C24'	1.0648 (15)	0.7630 (16)	0.2703 (7)	0.028 (3)	0.111 (2)
H24C	1.108807	0.827310	0.294428	0.034*	0.111 (2)
H24D	1.000941	0.763177	0.284495	0.034*	0.111 (2)
C25'	1.0439 (14)	0.7977 (14)	0.2021 (7)	0.029 (2)	0.111 (2)
C26'	1.0770 (16)	0.9117 (16)	0.1837 (8)	0.037 (3)	0.111 (2)
H26'	1.114195	0.966922	0.213816	0.044*	0.111 (2)
C27'	1.0559 (18)	0.944 (2)	0.1216 (9)	0.039 (3)	0.111 (2)
H27'	1.092008	1.010871	0.108094	0.047*	0.111 (2)
C28'	0.983 (2)	0.883 (2)	0.0789 (11)	0.037 (2)	0.111 (2)
H28'	0.952886	0.921149	0.040544	0.045*	0.111 (2)
C29'	0.9555 (19)	0.762 (2)	0.0934 (10)	0.032 (3)	0.111 (2)
H29'	0.913261	0.711548	0.063023	0.038*	0.111 (2)
C30'	0.992 (4)	0.717 (3)	0.1543 (11)	0.025 (2)	0.111 (2)
C31'	0.958 (3)	0.594 (3)	0.1700 (16)	0.0178 (7)	0.111 (2)
H31'	0.909606	0.556218	0.138068	0.021*	0.111 (2)
C32'	1.2026 (13)	0.6327 (17)	0.2576 (10)	0.034 (4)	0.111 (2)
H32D	1.233756	0.550496	0.266794	0.052*	0.111 (2)
H32E	1.184002	0.644708	0.212440	0.052*	0.111 (2)
H32F	1.250184	0.697811	0.276145	0.052*	0.111 (2)
C33'	1.1427 (16)	0.6235 (17)	0.3521 (8)	0.031 (2)	0.111 (2)
H33C	1.204865	0.672026	0.367182	0.037*	0.111 (2)
H33D	1.090134	0.661197	0.370847	0.037*	0.111 (2)
C34'	1.160 (2)	0.4947 (19)	0.3763 (11)	0.038 (3)	0.111 (2)
H34C	1.098900	0.459873	0.386468	0.046*	0.111 (2)
H34D	1.181367	0.439951	0.345226	0.046*	0.111 (2)
O1'	1.2380 (19)	0.5042 (18)	0.4307 (11)	0.040 (3)	0.111 (2)
C35'	1.253 (2)	0.391 (2)	0.4642 (12)	0.053 (5)	0.111 (2)
H35D	1.306612	0.402033	0.501224	0.080*	0.111 (2)
H35E	1.190802	0.368067	0.476824	0.080*	0.111 (2)
H35F	1.271099	0.325597	0.437849	0.080*	0.111 (2)

### Atomic displacement parameters (Å^2^)

**Table:**

	*U*^11^	*U*^22^	*U*^33^	*U*^12^	*U*^13^	*U*^23^
Ru1	0.01335 (7)	0.01573 (7)	0.01514 (7)	0.00000 (6)	0.00443 (5)	−0.00009 (6)
Cl1	0.0265 (2)	0.0272 (2)	0.0246 (2)	0.00392 (19)	0.01267 (19)	−0.00192 (19)
Cl2	0.0183 (4)	0.0286 (5)	0.0318 (4)	0.0050 (3)	0.0098 (3)	−0.0013 (3)
Cl2'	0.0183 (4)	0.0286 (5)	0.0318 (4)	0.0050 (3)	0.0098 (3)	−0.0013 (3)
N1	0.0259 (8)	0.0156 (7)	0.0162 (8)	−0.0041 (6)	0.0065 (6)	−0.0008 (6)
N3	0.0259 (9)	0.0166 (8)	0.0167 (8)	−0.0034 (6)	0.0062 (6)	0.0022 (6)
C2	0.0156 (8)	0.0193 (9)	0.0169 (9)	0.0008 (7)	0.0079 (7)	0.0010 (7)
C4	0.0408 (13)	0.0188 (10)	0.0259 (11)	−0.0061 (9)	0.0057 (9)	0.0018 (8)
C5	0.0372 (12)	0.0172 (9)	0.0226 (10)	−0.0085 (8)	0.0095 (8)	−0.0021 (7)
C6	0.0280 (10)	0.0170 (9)	0.0136 (8)	−0.0043 (7)	0.0051 (7)	−0.0011 (7)
C7	0.0329 (11)	0.0217 (10)	0.0191 (10)	−0.0029 (8)	0.0090 (8)	−0.0027 (7)
C8	0.0375 (12)	0.0303 (11)	0.0186 (10)	−0.0058 (9)	0.0119 (8)	−0.0010 (8)
C9	0.0374 (11)	0.0270 (10)	0.0163 (9)	−0.0079 (10)	0.0029 (8)	−0.0010 (8)
C10	0.0283 (10)	0.0266 (10)	0.0182 (9)	−0.0010 (9)	−0.0004 (7)	−0.0007 (8)
C11	0.0255 (10)	0.0227 (10)	0.0153 (9)	−0.0063 (8)	0.0041 (7)	−0.0042 (7)
C12	0.0410 (13)	0.0385 (13)	0.0289 (11)	0.0087 (10)	0.0206 (10)	0.0043 (9)
C13	0.0520 (15)	0.0416 (14)	0.0220 (11)	−0.0020 (11)	0.0032 (10)	0.0080 (10)
C14	0.0245 (10)	0.0374 (12)	0.0193 (10)	0.0006 (9)	0.0062 (8)	−0.0014 (8)
C15	0.0256 (10)	0.0204 (9)	0.0167 (9)	−0.0036 (8)	0.0061 (7)	0.0036 (7)
C16	0.0258 (10)	0.0249 (10)	0.0212 (10)	−0.0053 (8)	0.0078 (8)	0.0037 (8)
C17	0.0349 (12)	0.0379 (12)	0.0226 (10)	−0.0018 (10)	0.0126 (9)	0.0031 (9)
C18	0.0419 (13)	0.0426 (13)	0.0198 (10)	−0.0013 (11)	0.0070 (9)	0.0036 (9)
C19	0.0341 (12)	0.0388 (13)	0.0231 (11)	0.0041 (10)	−0.0014 (9)	0.0100 (9)
C20	0.0302 (11)	0.0259 (10)	0.0238 (10)	0.0020 (8)	0.0070 (8)	0.0064 (8)
C21	0.0246 (11)	0.0367 (12)	0.0277 (11)	−0.0054 (9)	0.0087 (8)	0.0028 (9)
C22	0.0585 (18)	0.084 (2)	0.0208 (12)	0.0085 (15)	0.0064 (12)	0.0079 (13)
C23	0.0353 (13)	0.0426 (13)	0.0358 (13)	0.0141 (11)	0.0114 (10)	0.0124 (10)
N2	0.0206 (11)	0.0230 (12)	0.0281 (11)	−0.0029 (9)	0.0018 (8)	−0.0048 (9)
C24	0.0252 (12)	0.0284 (12)	0.0416 (14)	−0.0114 (10)	0.0113 (10)	−0.0011 (10)
C25	0.0327 (13)	0.0228 (11)	0.0363 (13)	−0.0056 (10)	0.0178 (10)	0.0006 (10)
C26	0.0494 (16)	0.0289 (13)	0.0576 (18)	−0.0106 (12)	0.0256 (14)	0.0059 (12)
C27	0.070 (2)	0.0288 (15)	0.058 (2)	−0.0015 (15)	0.0365 (18)	0.0157 (14)
C28	0.063 (2)	0.0300 (15)	0.0356 (17)	0.0111 (14)	0.0279 (14)	0.0118 (13)
C29	0.0398 (16)	0.0235 (11)	0.0250 (12)	0.0070 (12)	0.0191 (12)	0.0043 (9)
C30	0.0277 (19)	0.0165 (12)	0.0273 (14)	0.0006 (10)	0.0138 (12)	0.0003 (11)
C31	0.0166 (17)	0.0179 (9)	0.0202 (9)	−0.0001 (10)	0.0066 (11)	−0.0014 (7)
C32	0.0332 (14)	0.0194 (11)	0.0349 (14)	−0.0032 (10)	0.0027 (11)	−0.0090 (10)
C33	0.0214 (12)	0.0338 (13)	0.0362 (14)	−0.0044 (10)	−0.0046 (10)	−0.0061 (11)
C34	0.0298 (14)	0.0379 (16)	0.0325 (14)	−0.0016 (13)	−0.0071 (11)	−0.0045 (13)
O1	0.0465 (14)	0.0516 (17)	0.0440 (12)	−0.0089 (14)	−0.0216 (10)	0.0068 (14)
C35	0.067 (2)	0.069 (3)	0.048 (2)	−0.016 (2)	−0.0226 (18)	0.0153 (18)
N2'	0.025 (4)	0.027 (4)	0.031 (4)	−0.007 (4)	0.008 (4)	−0.006 (4)
C24'	0.028 (5)	0.025 (5)	0.032 (5)	−0.009 (4)	0.009 (4)	−0.006 (5)
C25'	0.034 (4)	0.021 (4)	0.037 (4)	−0.009 (4)	0.015 (4)	0.005 (4)
C26'	0.045 (5)	0.025 (5)	0.044 (5)	−0.008 (5)	0.017 (5)	0.005 (5)
C27'	0.053 (5)	0.026 (4)	0.045 (5)	0.000 (4)	0.025 (5)	0.009 (4)
C28'	0.051 (4)	0.026 (4)	0.040 (4)	−0.003 (4)	0.025 (4)	0.012 (4)
C29'	0.041 (5)	0.025 (4)	0.034 (4)	0.000 (5)	0.017 (5)	0.000 (4)
C30'	0.030 (4)	0.018 (4)	0.028 (4)	0.002 (4)	0.010 (4)	0.004 (4)
C31'	0.0166 (17)	0.0179 (9)	0.0202 (9)	−0.0001 (10)	0.0066 (11)	−0.0014 (7)
C32'	0.028 (7)	0.020 (7)	0.054 (8)	−0.008 (6)	0.005 (7)	−0.002 (7)
C33'	0.027 (4)	0.031 (4)	0.030 (4)	−0.010 (4)	−0.006 (4)	−0.006 (4)
C34'	0.035 (5)	0.037 (5)	0.035 (4)	−0.007 (5)	−0.008 (4)	0.001 (5)
O1'	0.034 (5)	0.041 (6)	0.036 (5)	−0.008 (5)	−0.014 (4)	0.008 (5)
C35'	0.048 (10)	0.053 (10)	0.045 (9)	−0.017 (9)	−0.018 (8)	0.023 (9)

### Geometric parameters (Å, º)

**Table:**

Ru1—C31'	1.81 (3)	N2—C33	1.501 (3)
Ru1—C31	1.833 (4)	C24—C25	1.497 (3)
Ru1—C2	2.0474 (18)	C24—H24A	0.9900
Ru1—N2'	2.251 (15)	C24—H24B	0.9900
Ru1—N2	2.271 (2)	C25—C26	1.393 (3)
Ru1—Cl2	2.3515 (8)	C25—C30	1.421 (5)
Ru1—Cl1	2.3519 (5)	C26—C27	1.383 (5)
Ru1—Cl2'	2.379 (7)	C26—H26	0.9500
N1—C2	1.352 (2)	C27—C28	1.382 (5)
N1—C6	1.434 (2)	C27—H27	0.9500
N1—C5	1.478 (2)	C28—C29	1.388 (4)
N3—C2	1.350 (2)	C28—H28	0.9500
N3—C15	1.441 (2)	C29—C30	1.401 (4)
N3—C4	1.476 (2)	C29—H29	0.9500
C4—C5	1.518 (3)	C30—C31	1.462 (3)
C4—H4A	0.9900	C31—H31	0.9500
C4—H4B	0.9900	C32—H32A	0.9800
C5—H5A	0.9900	C32—H32B	0.9800
C5—H5B	0.9900	C32—H32C	0.9800
C6—C11	1.396 (3)	C33—C34	1.511 (4)
C6—C7	1.401 (3)	C33—H33A	0.9900
C7—C8	1.400 (3)	C33—H33B	0.9900
C7—C12	1.502 (3)	C34—O1	1.420 (3)
C8—C9	1.384 (3)	C34—H34A	0.9900
C8—H8	0.9500	C34—H34B	0.9900
C9—C10	1.393 (3)	O1—C35	1.408 (4)
C9—C13	1.508 (3)	C35—H35A	0.9800
C10—C11	1.390 (3)	C35—H35B	0.9800
C10—H10	0.9500	C35—H35C	0.9800
C11—C14	1.505 (3)	N2'—C24'	1.465 (17)
C12—H12A	0.9800	N2'—C32'	1.472 (18)
C12—H12B	0.9800	N2'—C33'	1.486 (16)
C12—H12C	0.9800	C24'—C25'	1.514 (15)
C13—H13A	0.9800	C24'—H24C	0.9900
C13—H13B	0.9800	C24'—H24D	0.9900
C13—H13C	0.9800	C25'—C26'	1.393 (15)
C14—H14A	0.9800	C25'—C30'	1.423 (17)
C14—H14B	0.9800	C26'—C27'	1.380 (17)
C14—H14C	0.9800	C26'—H26'	0.9500
C15—C20	1.397 (3)	C27'—C28'	1.379 (18)
C15—C16	1.401 (3)	C27'—H27'	0.9500
C16—C17	1.391 (3)	C28'—C29'	1.407 (17)
C16—C21	1.501 (3)	C28'—H28'	0.9500
C17—C18	1.391 (3)	C29'—C30'	1.410 (16)
C17—H17	0.9500	C29'—H29'	0.9500
C18—C19	1.379 (3)	C30'—C31'	1.465 (17)
C18—C22	1.513 (3)	C31'—H31'	0.9500
C19—C20	1.396 (3)	C32'—H32D	0.9800
C19—H19	0.9500	C32'—H32E	0.9800
C20—C23	1.511 (3)	C32'—H32F	0.9800
C21—H21A	0.9800	C33'—C34'	1.480 (16)
C21—H21B	0.9800	C33'—H33C	0.9900
C21—H21C	0.9800	C33'—H33D	0.9900
C22—H22A	0.9800	C34'—O1'	1.421 (17)
C22—H22B	0.9800	C34'—H34C	0.9900
C22—H22C	0.9800	C34'—H34D	0.9900
C23—H23A	0.9800	O1'—C35'	1.407 (17)
C23—H23B	0.9800	C35'—H35D	0.9800
C23—H23C	0.9800	C35'—H35E	0.9800
N2—C32	1.489 (3)	C35'—H35F	0.9800
N2—C24	1.491 (4)		
			
C31'—Ru1—C2	100.5 (8)	C24—N2—C33	105.1 (2)
C31—Ru1—C2	97.33 (11)	C32—N2—Ru1	115.62 (17)
C31'—Ru1—N2'	92.1 (8)	C24—N2—Ru1	108.54 (16)
C2—Ru1—N2'	163.8 (5)	C33—N2—Ru1	110.59 (15)
C31—Ru1—N2	88.48 (11)	N2—C24—C25	113.7 (2)
C2—Ru1—N2	172.68 (8)	N2—C24—H24A	108.8
C31—Ru1—Cl2	103.42 (13)	C25—C24—H24A	108.8
C2—Ru1—Cl2	86.77 (5)	N2—C24—H24B	108.8
N2—Ru1—Cl2	87.55 (7)	C25—C24—H24B	108.8
C31'—Ru1—Cl1	104.7 (14)	H24A—C24—H24B	107.7
C31—Ru1—Cl1	98.02 (13)	C26—C25—C30	118.8 (3)
C2—Ru1—Cl1	95.36 (5)	C26—C25—C24	119.0 (2)
N2'—Ru1—Cl1	91.3 (6)	C30—C25—C24	122.2 (2)
N2—Ru1—Cl1	88.21 (7)	C27—C26—C25	121.8 (3)
Cl2—Ru1—Cl1	158.02 (3)	C27—C26—H26	119.1
C31'—Ru1—Cl2'	96.9 (15)	C25—C26—H26	119.1
C2—Ru1—Cl2'	78.3 (2)	C28—C27—C26	119.8 (3)
N2'—Ru1—Cl2'	90.2 (6)	C28—C27—H27	120.1
Cl1—Ru1—Cl2'	158.4 (3)	C26—C27—H27	120.1
C2—N1—C6	128.05 (15)	C27—C28—C29	119.6 (3)
C2—N1—C5	114.09 (15)	C27—C28—H28	120.2
C6—N1—C5	117.11 (15)	C29—C28—H28	120.2
C2—N3—C15	126.88 (16)	C28—C29—C30	121.8 (3)
C2—N3—C4	114.39 (15)	C28—C29—H29	119.1
C15—N3—C4	117.90 (15)	C30—C29—H29	119.1
N3—C2—N1	106.27 (15)	C29—C30—C25	118.2 (3)
N3—C2—Ru1	121.14 (13)	C29—C30—C31	117.5 (3)
N1—C2—Ru1	131.33 (13)	C25—C30—C31	124.3 (3)
N3—C4—C5	102.46 (15)	C30—C31—Ru1	130.7 (2)
N3—C4—H4A	111.3	C30—C31—H31	114.7
C5—C4—H4A	111.3	Ru1—C31—H31	114.7
N3—C4—H4B	111.3	N2—C32—H32A	109.5
C5—C4—H4B	111.3	N2—C32—H32B	109.5
H4A—C4—H4B	109.2	H32A—C32—H32B	109.5
N1—C5—C4	102.64 (15)	N2—C32—H32C	109.5
N1—C5—H5A	111.2	H32A—C32—H32C	109.5
C4—C5—H5A	111.2	H32B—C32—H32C	109.5
N1—C5—H5B	111.2	N2—C33—C34	113.4 (2)
C4—C5—H5B	111.2	N2—C33—H33A	108.9
H5A—C5—H5B	109.2	C34—C33—H33A	108.9
C11—C6—C7	121.84 (17)	N2—C33—H33B	108.9
C11—C6—N1	118.94 (16)	C34—C33—H33B	108.9
C7—C6—N1	119.16 (17)	H33A—C33—H33B	107.7
C8—C7—C6	117.70 (19)	O1—C34—C33	106.3 (2)
C8—C7—C12	120.82 (18)	O1—C34—H34A	110.5
C6—C7—C12	121.48 (18)	C33—C34—H34A	110.5
C9—C8—C7	121.88 (19)	O1—C34—H34B	110.5
C9—C8—H8	119.1	C33—C34—H34B	110.5
C7—C8—H8	119.1	H34A—C34—H34B	108.7
C8—C9—C10	118.54 (18)	C35—O1—C34	112.1 (3)
C8—C9—C13	121.43 (19)	O1—C35—H35A	109.5
C10—C9—C13	120.0 (2)	O1—C35—H35B	109.5
C11—C10—C9	121.89 (19)	H35A—C35—H35B	109.5
C11—C10—H10	119.1	O1—C35—H35C	109.5
C9—C10—H10	119.1	H35A—C35—H35C	109.5
C10—C11—C6	118.04 (18)	H35B—C35—H35C	109.5
C10—C11—C14	120.70 (18)	C24'—N2'—C32'	110.3 (15)
C6—C11—C14	121.18 (17)	C24'—N2'—C33'	109.1 (15)
C7—C12—H12A	109.5	C32'—N2'—C33'	108.5 (15)
C7—C12—H12B	109.5	C24'—N2'—Ru1	105.1 (11)
H12A—C12—H12B	109.5	C32'—N2'—Ru1	112.5 (13)
C7—C12—H12C	109.5	C33'—N2'—Ru1	111.4 (12)
H12A—C12—H12C	109.5	N2'—C24'—C25'	113.5 (15)
H12B—C12—H12C	109.5	N2'—C24'—H24C	108.9
C9—C13—H13A	109.5	C25'—C24'—H24C	108.9
C9—C13—H13B	109.5	N2'—C24'—H24D	108.9
H13A—C13—H13B	109.5	C25'—C24'—H24D	108.9
C9—C13—H13C	109.5	H24C—C24'—H24D	107.7
H13A—C13—H13C	109.5	C26'—C25'—C30'	117.1 (15)
H13B—C13—H13C	109.5	C26'—C25'—C24'	120.1 (14)
C11—C14—H14A	109.5	C30'—C25'—C24'	122.8 (14)
C11—C14—H14B	109.5	C27'—C26'—C25'	120.0 (16)
H14A—C14—H14B	109.5	C27'—C26'—H26'	120.0
C11—C14—H14C	109.5	C25'—C26'—H26'	120.0
H14A—C14—H14C	109.5	C28'—C27'—C26'	121.3 (17)
H14B—C14—H14C	109.5	C28'—C27'—H27'	119.3
C20—C15—C16	121.73 (17)	C26'—C27'—H27'	119.3
C20—C15—N3	119.80 (17)	C27'—C28'—C29'	118.4 (18)
C16—C15—N3	118.11 (17)	C27'—C28'—H28'	120.8
C17—C16—C15	117.70 (19)	C29'—C28'—H28'	120.8
C17—C16—C21	120.32 (18)	C28'—C29'—C30'	118.0 (18)
C15—C16—C21	121.95 (17)	C28'—C29'—H29'	121.0
C16—C17—C18	122.0 (2)	C30'—C29'—H29'	121.0
C16—C17—H17	119.0	C29'—C30'—C25'	121.1 (17)
C18—C17—H17	119.0	C29'—C30'—C31'	117.7 (18)
C19—C18—C17	118.4 (2)	C25'—C30'—C31'	120.1 (19)
C19—C18—C22	120.9 (2)	C30'—C31'—Ru1	130.8 (19)
C17—C18—C22	120.6 (2)	C30'—C31'—H31'	114.6
C18—C19—C20	122.1 (2)	Ru1—C31'—H31'	114.6
C18—C19—H19	118.9	N2'—C32'—H32D	109.5
C20—C19—H19	118.9	N2'—C32'—H32E	109.5
C19—C20—C15	117.78 (19)	H32D—C32'—H32E	109.5
C19—C20—C23	120.05 (19)	N2'—C32'—H32F	109.5
C15—C20—C23	122.17 (18)	H32D—C32'—H32F	109.5
C16—C21—H21A	109.5	H32E—C32'—H32F	109.5
C16—C21—H21B	109.5	C34'—C33'—N2'	117.9 (16)
H21A—C21—H21B	109.5	C34'—C33'—H33C	107.8
C16—C21—H21C	109.5	N2'—C33'—H33C	107.8
H21A—C21—H21C	109.5	C34'—C33'—H33D	107.8
H21B—C21—H21C	109.5	N2'—C33'—H33D	107.8
C18—C22—H22A	109.5	H33C—C33'—H33D	107.2
C18—C22—H22B	109.5	O1'—C34'—C33'	105.7 (15)
H22A—C22—H22B	109.5	O1'—C34'—H34C	110.6
C18—C22—H22C	109.5	C33'—C34'—H34C	110.6
H22A—C22—H22C	109.5	O1'—C34'—H34D	110.6
H22B—C22—H22C	109.5	C33'—C34'—H34D	110.6
C20—C23—H23A	109.5	H34C—C34'—H34D	108.7
C20—C23—H23B	109.5	C35'—O1'—C34'	112.1 (17)
H23A—C23—H23B	109.5	O1'—C35'—H35D	109.5
C20—C23—H23C	109.5	O1'—C35'—H35E	109.5
H23A—C23—H23C	109.5	H35D—C35'—H35E	109.5
H23B—C23—H23C	109.5	O1'—C35'—H35F	109.5
C32—N2—C24	107.9 (2)	H35D—C35'—H35F	109.5
C32—N2—C33	108.6 (2)	H35E—C35'—H35F	109.5
			
C15—N3—C2—N1	−167.34 (17)	C33—N2—C24—C25	−176.1 (2)
C4—N3—C2—N1	1.9 (2)	Ru1—N2—C24—C25	65.6 (2)
C15—N3—C2—Ru1	24.1 (2)	N2—C24—C25—C26	138.3 (2)
C4—N3—C2—Ru1	−166.63 (14)	N2—C24—C25—C30	−42.9 (4)
C6—N1—C2—N3	170.61 (17)	C30—C25—C26—C27	−1.6 (5)
C5—N1—C2—N3	0.9 (2)	C24—C25—C26—C27	177.2 (3)
C6—N1—C2—Ru1	−22.5 (3)	C25—C26—C27—C28	0.3 (5)
C5—N1—C2—Ru1	167.80 (14)	C26—C27—C28—C29	0.5 (5)
C2—N3—C4—C5	−3.7 (2)	C27—C28—C29—C30	0.2 (5)
C15—N3—C4—C5	166.58 (17)	C28—C29—C30—C25	−1.5 (6)
C2—N1—C5—C4	−3.1 (2)	C28—C29—C30—C31	179.1 (4)
C6—N1—C5—C4	−174.01 (16)	C26—C25—C30—C29	2.2 (6)
N3—C4—C5—N1	3.7 (2)	C24—C25—C30—C29	−176.6 (3)
C2—N1—C6—C11	−78.7 (2)	C26—C25—C30—C31	−178.5 (4)
C5—N1—C6—C11	90.8 (2)	C24—C25—C30—C31	2.8 (7)
C2—N1—C6—C7	104.2 (2)	C29—C30—C31—Ru1	−178.9 (3)
C5—N1—C6—C7	−86.3 (2)	C25—C30—C31—Ru1	1.7 (8)
C11—C6—C7—C8	2.9 (3)	C2—Ru1—C31—C30	−156.7 (4)
N1—C6—C7—C8	179.88 (17)	N2—Ru1—C31—C30	18.9 (5)
C11—C6—C7—C12	−177.59 (19)	Cl2—Ru1—C31—C30	−68.3 (5)
N1—C6—C7—C12	−0.6 (3)	Cl1—Ru1—C31—C30	106.8 (4)
C6—C7—C8—C9	−0.3 (3)	C32—N2—C33—C34	78.3 (3)
C12—C7—C8—C9	−179.8 (2)	C24—N2—C33—C34	−166.5 (2)
C7—C8—C9—C10	−2.2 (3)	Ru1—N2—C33—C34	−49.6 (3)
C7—C8—C9—C13	176.8 (2)	N2—C33—C34—O1	−162.1 (2)
C8—C9—C10—C11	2.2 (3)	C33—C34—O1—C35	−169.0 (3)
C13—C9—C10—C11	−176.80 (19)	C32'—N2'—C24'—C25'	53 (2)
C9—C10—C11—C6	0.3 (3)	C33'—N2'—C24'—C25'	172.0 (16)
C9—C10—C11—C14	177.06 (18)	Ru1—N2'—C24'—C25'	−68.4 (17)
C7—C6—C11—C10	−2.9 (3)	N2'—C24'—C25'—C26'	−128 (2)
N1—C6—C11—C10	−179.88 (16)	N2'—C24'—C25'—C30'	51 (4)
C7—C6—C11—C14	−179.65 (18)	C30'—C25'—C26'—C27'	2 (4)
N1—C6—C11—C14	3.3 (3)	C24'—C25'—C26'—C27'	−178 (2)
C2—N3—C15—C20	−105.2 (2)	C25'—C26'—C27'—C28'	16 (4)
C4—N3—C15—C20	85.9 (2)	C26'—C27'—C28'—C29'	−22 (5)
C2—N3—C15—C16	81.5 (2)	C27'—C28'—C29'—C30'	9 (5)
C4—N3—C15—C16	−87.4 (2)	C28'—C29'—C30'—C25'	9 (7)
C20—C15—C16—C17	5.4 (3)	C28'—C29'—C30'—C31'	177 (4)
N3—C15—C16—C17	178.51 (18)	C26'—C25'—C30'—C29'	−15 (6)
C20—C15—C16—C21	−172.71 (19)	C24'—C25'—C30'—C29'	166 (3)
N3—C15—C16—C21	0.4 (3)	C26'—C25'—C30'—C31'	178 (4)
C15—C16—C17—C18	−2.5 (3)	C24'—C25'—C30'—C31'	−1 (7)
C21—C16—C17—C18	175.7 (2)	C29'—C30'—C31'—Ru1	175 (3)
C16—C17—C18—C19	−1.5 (3)	C25'—C30'—C31'—Ru1	−16 (8)
C16—C17—C18—C22	179.6 (2)	C2—Ru1—C31'—C30'	−174 (5)
C17—C18—C19—C20	2.7 (4)	N2'—Ru1—C31'—C30'	−5 (5)
C22—C18—C19—C20	−178.4 (2)	Cl1—Ru1—C31'—C30'	87 (5)
C18—C19—C20—C15	0.1 (3)	Cl2'—Ru1—C31'—C30'	−95 (5)
C18—C19—C20—C23	−179.1 (2)	C24'—N2'—C33'—C34'	160 (2)
C16—C15—C20—C19	−4.2 (3)	C32'—N2'—C33'—C34'	−80 (2)
N3—C15—C20—C19	−177.24 (18)	Ru1—N2'—C33'—C34'	44 (2)
C16—C15—C20—C23	174.9 (2)	N2'—C33'—C34'—O1'	147 (2)
N3—C15—C20—C23	1.9 (3)	C33'—C34'—O1'—C35'	171 (3)
C32—N2—C24—C25	−60.4 (3)		

### Hydrogen-bond geometry (Å, º)

**Table:**

*D*—H···*A*	*D*—H	H···*A*	*D*···*A*	*D*—H···*A*
C4—H4*A*···Cl1^i^	0.99	2.89	3.651 (2)	134
C5—H5*B*···Cl1^i^	0.99	2.83	3.622 (2)	137
C24—H24*A*···Cl2	0.99	2.56	3.257 (3)	127
C24—H24*B*···Cl2^ii^	0.99	2.72	3.693 (3)	167
C33—H33*B*···Cl2	0.99	2.87	3.399 (3)	114
C34—H34*A*···Cl1	0.99	2.94	3.570 (3)	123
C24′—H24*D*···Cl1	0.99	2.68	3.391 (19)	129
C32′—H32*D*···Cl2′	0.98	2.59	3.07 (2)	110
C32′—H32*F*···Cl2′^ii^	0.98	2.62	3.57 (2)	164
C4—H4*A*···Cl1^i^	0.99	2.89	3.651 (2)	134
C5—H5*B*···Cl1^i^	0.99	2.83	3.622 (2)	137
C24—H24*A*···Cl2	0.99	2.56	3.257 (3)	127
C24—H24*B*···Cl2^ii^	0.99	2.72	3.693 (3)	167
C33—H33*B*···Cl2	0.99	2.87	3.399 (3)	114
C34—H34*A*···Cl1	0.99	2.94	3.570 (3)	123
C24′—H24*D*···Cl1	0.99	2.68	3.391 (19)	129
C32′—H32*D*···Cl2′	0.98	2.59	3.07 (2)	110
C32′—H32*F*···Cl2′^ii^	0.98	2.62	3.57 (2)	164
C34′—H34*C*···*Cg*3	0.99	2.96	3.83 (2)	149

Symmetry codes: (i) −*x*+3/2, *y*−1/2, −*z*+1/2; (ii) −*x*+5/2, *y*+1/2, −*z*+1/2.
